# Relational trust in outreach with women experiencing street-involvement in British Columbia, Canada: a qualitative study

**DOI:** 10.1186/s12913-025-13875-3

**Published:** 2025-12-11

**Authors:** Michelle Gagnon, Sunny Jiao, Shahin Kassam, Linda Dewar, Patricia Tait, Vicky Bungay

**Affiliations:** 1 Capacity: The Centre for Research in Community Engagement and Gender Equity, School of Nursing, University of British Columbia, Room 5280 – 5th Floor, 5955 University Blvd, Vancouver, BC V6T 1Z1 Canada; 2 Inner-City Women’s Initiatives Society, 101 E Cordova St, Vancouver, BC V6A 1K7 Canada

**Keywords:** Women, Violence, Gender, Substance use, Poverty, Outreach, Access to care

## Abstract

**Background:**

Women who experience street-involvement are severely underserved in health care. Outreach can address this service gap, but little is known about what constitutes gender-appropriate and effective outreach worker-client relationships in this context.

**Methods:**

To explore the relational attributes of outreach, qualitative interviews were conducted with women (*n* = 19) enrolled in a pilot study (2017–2020) aimed at designing a gender-specific outreach program in British Columbia and with outreach staff (*n* = 8). Data analysis focused on women’s experiences, supplemented by outreach staffs’ perspectives. Reflexive thematic analysis informed by central tenets of feminist relational theory and harm reduction was used.

**Results:**

Trust was the overarching theme, conceptualized as a relational, dynamic, and iterative process co-constructed over time. Eight interrelated domains of trust were identified: commitment; consistency; professional boundaries; privacy and confidentiality; empathy; non-judgement; expert knowledge; and “doing with not for”. Relational trust integrated understandings of the impact of gender-based violence, poverty, and drug-related criminalization. When enacted, trust facilitated participants’ engagement with outreach workers, improved care access, and advanced women’s capacities to navigate their general and substance use health care more independently.

**Conclusions:**

Trust is a critical element of health and social service delivery and distrust remains a major barrier. These findings advance new conceptualizations of trust that consider the interplay between interpersonal and structural features of care delivery such as stigma, discrimination, colonization, ableism, and classism. Outreach programs emphasizing trust-building as a dynamic and socio-structurally embedded process hold significant potential for improving care engagement among women who are chronically underserved.

**Trial registration:**

Retrospectively registered on February 25, 2025 (NCT06854770) with ClinicalTrials.gov.

**Supplementary Information:**

The online version contains supplementary material available at 10.1186/s12913-025-13875-3.

## Background

Women experiencing street-involvement face substantial barriers to appropriate and timely health and social care; a situation that directly exacerbates their health inequities and premature death [1, 2]. For our purposes, street-involvement is a broad term associated with processes of significant structural disadvantage including but not limited to poverty, inadequate shelter, high degrees of public visibility and state control (i.e., criminalization associated with survival strategies), disproportionate burden of chronic health issues, inequitable receipt of health services aligned with personal needs, and stigma and discrimination [3]. Research to address barriers to care among women experiencing street-involvement has historically prioritized women’s treatment adherence, care attendance and care avoidance. Such research is often underpinned by misperceptions concerning women’s capacities to seek help and/or their reluctance to engage with care due to what has been termed their *‘chaotic lifestyle’* [4, 6]. Consequently, women experiencing street-involvement are often described in research and care settings as a hidden or hard to reach group [3–5]. Feminist and intersectional scholars have challenged this micro-individualistic notion of hidden or hard to reach, noting the importance of addressing the multiple and intersecting structural conditions (e.g., colonialism, racism, sexism, classism, criminalization, and gender-based violence) shaping how care is organized and delivered and women’s engagement therein [4, 6–13]. For example, women who use unregulated drugs – a common situation for those experiencing street-involvement [1–4], are regularly seen within care encounters as transgressing normative gender stereotypes contributing to significant stigma and discrimination including being infantilized as victims lacking agency and/or considered as inherently more pathological and criminal than men who use drugs [4, 7, 9, 14]. Care spaces and programming such as housing, health care, harm reduction, and drug treatment and recovery services are also problematic [9, 15–20]. Many of these services for instance, are devoid of gender-specific considerations endemic to women with lived/living experience of street-involvement, including gender-based violence and the feminization of poverty that directly impact the accessibility and appropriateness of services [9, 15–17]. For example, women experiencing street-involvement are often forced to attend to co-ed spaces despite evidence supporting the risk of violence within these spaces [15–17]. Additionally, as noted earlier, women report significant stigma in care encounters related to transgressing what is perceived as appropriate gender-roles [4, 7, 9, 14]. Accordingly, women are chronically underserved contributing to unmet care needs [2, 11, 18, 19]; reliance on emergency care services [4, 20]; and their disproportionate burden of chronic illnesses, poverty, ongoing violence, and premature death [1].

Outreach – defined as the relational processes and practices to locate and make connections with people chronically underserved in health and social care [3, 4] – is an established strategy to enhance care engagement among diverse populations experiencing street-involvement including women and other people who use unregulated drugs, and among those experiencing homelessness [21], mental health challenges [22] and engaged in survival sex work [23, 24]. While there is no standardized outreach model of care, outreach programs are regularly considered low-barrier as a means to facilitate engagement and service uptake [25]. For example, outreach programs may offer unconditional episodic supports including harm reduction supplies and other overdose prevention services, food and water, and emergency assistance for shelter or health care without expectation of enrollment or engagement in other programs or services [25]. Outreach also occurs in community spaces where people spend their time such as parks, drop-in centres, encampments, on the street, and in people’s homes and emergency shelters thereby garnering the phrase “meeting people where they are at” [25].

Today, in Canada, and many other parts of the world, women experiencing street-involvement continue to experience the ongoing devastation associated with the unregulated drug poisoning crisis [8, 15, 16]. Unregulated drugs are those produced and distributed without quality control and oversight leading to unpredictable potency that exacerbates significant health risks [26]. We use the term unregulated instead of illicit to avoid potentially moralistic overtones that overlook the structural context exacerbating drug related harms. This crisis is worsened by increasing public health and police surveillance and accompanying policy changes that limit harm reduction interventions such as the operation of safe consumption spaces within people’s communities [27, 28]. Such policies have the direct results of worsening health and social inequities among those who use drugs and exacerbate mistrust in care and other systemic barriers [27]. As a means to address these inequities and enhance service delivery, outreach programs with people who use drugs are on the rise [25]. This expansion has been accompanied by a growing interest in outreach effectiveness to facilitate care access and the relational elements of outreach practice to achieve this aim [3, 4, 19, 22, 29]. In a recent systematic review [25] for instance, building and sustaining trust through outreach worker dependability and consistency and using respectful, strengths-based approaches to relationship building appear to be core elements of outreach implementation with women who use unregulated drugs [23, 30, 31]. Early research indicates that outreach in these contexts can support greater uptake of harm reduction supplies [31], improve women’s perceptions of safety [30], and enhance engagement with health care services [24, 32]. While such research is important, less attention has been paid to creating empirical understandings pertaining to the attributes of relational elements of trust (e.g., dependability, consistency), in addition to determining if and what other elements are relevant to the outreach worker-client relationship. Furthermore, what is known is largely from the perspective of outreach workers versus clients. The evidence base also remains largely gender-blind in that gender of clients is not considered in research, gender norms are ignored, or if norms are acknowledged, gender inequities inclusive of gender-based violence and related intersections with systemic racism, classism, criminalization, and ableism are not addressed [25].

This paper seeks to help address a gap in knowledge concerning the relational and gender-specific elements of outreach among women experiencing street-involvement by examining self-identifying women’s experiences of engaging in a pilot outreach intervention aimed at enhancing their access and receipt of health and social care. Our present analysis specifically seeks to identify the unique relational elements of outreach practice necessary to foster safe, respectful, and strengths-based relationships that consider the socio-structural context (e.g., poverty, racism, classism, ableism, sexism, drug-use, violence, stigma, discrimination and criminalization) shaping women’s history and ongoing relationships with health and social care.

## Methods

### Study context

In this article we draw on a sub-set of data collected as part of a 3-year, single case, pilot study known as the Sisters Together Reaching Every New Goal Towards Health (STRENGTH) Project, that aimed to design and test a novel, women-specific (transgender inclusive) outreach intervention in the Downtown Eastside (DTES) neighbourhood in British Columbia (BC), Canada. The DTES is among the poorest communities in Canada [33, 34] with death rates due to unregulated drug poisoning crisis 12 times the provincial average [35]. Almost 10% of these residents identify as Indigenous, compared to 2% across the entire city [36]; illustrating the impact of historical and ongoing colonization for Indigenous people. Women in the DTES are among those who have been murdered or gone missing in BC since the 1970’s; a direct result of serial killers targeting women experiencing street-involvement and a lack of societal response to this intentional, gendered violence [37, 38]. Although numerous health and social care services exist including those that are women-specific, women here continue to experience gendered barriers to care including unsafe co-ed spaces whereby women are at risk for violence by other clients in the space, and insufficient, gender and trauma-specific mental health care due to limited availability and appropriateness of such resources [33, 39].

Given the ongoing gender-based violence and inequities in health and in health and social care and the promising potential of outreach to improve care access, a research partnership was established among staff and leadership of the Inner-city Women’s Initiatives Society (ICWIS), women with lived/living experience of street-involvement, and researchers specializing in community-based and women’s health research. At the time of the study, ICWIS offered drop-in meal services and a case management program aimed at reducing women’s risk for HIV infection. Together, this team collaboratively co-designed an 18-month intervention, implemented by self-identifying female outreach workers trained in harm reduction and trauma- and violence-informed care principles (see Bungay et al., 2024 [3] for details). The outreach workers all had previous experience working with women experiencing street-involvement and expertise in health and social care navigation support. Outreach workers initiated connections through in-person, outreach in public settings such as parks or on the street and drop-in centres frequented by women. The initial interactions were low-barrier, offering food, harm reduction supplies, and emergency support for health care or shelter. As outreach workers became known to women, relationships built gradually over time whereby women enrolled to work 1–1 with outreach teams to collaboratively support participants in achieving their health and well-being goals, including navigating health and social services as needed. The intervention was grounded in theoretical and practice-based principles that addressed the impacts of gender-based violence, substance use related discrimination, and poverty on care engagement, emphasizing respect, reciprocity, harm reduction, and right to self-determination. While the process of developing and implementing these principles specific to outreach with self-identifying women are detailed elsewhere [3], a brief overview is provided in Table 1. Women, inclusive of cis and trans persons who self-identified as women, were eligible if they lived or spent significant time in the Downtown Eastside (DTES), faced barriers to accessing care, were aged 18 or older, and could communicate verbally in English (see Bungay et al., 2025 [4] for intervention implementation and outcomes).

**Table 1:** Guiding principles

Principle	Definition
Tackle gender-based violence	Gender-based violence is the harmful acts, practices, policies, and social stratifications that are directed at an individual or group based on their gender [4, 40–42]. Socio-structural facets of society contribute to and create conditions of interpersonal violence inclusive of physical, psychological and material acts of violence that are disproportionately experienced by women as a group, and particularly among those impacted by systemic inequities operating through racist, ableist, and class-based policy and practices [41].
Harm reduction as engagement	Harm reduction is a relational practice that emphasizes respect and non-judgment toward women’s use of unregulated substances while addressing the broader socio-structural context (e.g., poverty, violence, discrimination, criminalization) shaping their substance use and health opportunities [43].
Respectful and rights-based approach to personhood	Personhood encompasses people’s strengths, capacities, and inherent rights and capabilities to make their own choices. This requires a women-led approach to ascertaining their health concerns and strategies to mitigate these concerns.
Trauma and violence informed engagement (TVIE)	There are 6 core elements of TVIE: (1) it is vital that we understand that trauma has numerous impacts on women’s psychological and physical functioning; (2) women have the right to determine what constitutes safety and violence; (3) strengths-based approaches are critical to foster autonomy; (4) engagement with support workers should not cause harm; (5) a relational approach to engagement requires collaboration and continuous negotiation of expectations within the working relationship; (6) trauma is situated within intersectional causes [43, 44].

### Data collection

The subset of data used in this analysis included semi-structured one-hour interviews with enrolled STRENGTH participants (*n* = 19) and outreach staff (*n* = 8), intervention field notes (See Supplementary Material for Interview Guides), and a brief survey at enrollment to gather STRENGTH participant demographic characteristics and current access to health and social care. Outreach staff and managers were recruited from six community service organizations serving women experiencing homelessness, mental health, addictions and recovery, and post-incarceration transition. These interviews provided contextual information about current outreach services in the community including the nature of outreach work, barriers and facilitators to effective working relationships, and the context of women services in the DTES. All staff participants self-identified as women and had worked between 2.5 and 12 years in service delivery in the community. Semi-structured interviews were conducted during months 15–18 with a sub-set of enrolled STRENGTH participants who had at least two engagements with the outreach team. The subset was purposefully selected as they provided sufficient depth and breadth of their experiences with STRENGTH, including their perceptions of what worked well, what did not and why. All interviews were conducted by VB or a research staff member at a place considered safe and appropriate to the participant (i.e., a private space in a trusted service organization, participant homes) and interview guides were developed specifically for the study. Informed consent was obtained verbally and recorded electronically using REDCap electronic data capture tools [45, 46]. In keeping with recommendations of women with lived/living experience within the DTES who were as noted previously, involved in the design of the study [3], STRENGTH participants received $25 honoraria prior to the interview as incentive to participate and in recognition of their time. Interviews were audio recorded, transcribed and checked for accuracy by research staff, anonymized and uploaded to NVivo, a qualitative data management program to facilitate data analysis. Field notes were collected throughout the 18-month intervention period and included a bi-weekly reflective journal completed by outreach workers at the end of each week (*n* = 70 entries) noting the strengths and challenges in building relationships with participants. Monthly team and community advisory meetings’ decisions to address challenges in intervention implementation were also included. Field note data were entered as open text and downloaded into Excel. The research was conducted in accordance with the standards and considerations set out in the Canadian Tri-Council Policy Statement: Ethical Conduct for Research Involving Humans (TCPS 2) and the Declaration of Helsinki. The study received ethics approval from the University of British Columbia Behavioural Ethics Review Board, Ethics Certificate Number: H18-00069.

### Analytical perspectives

Feminist relational theory posits that all individuals are inherently social beings who are influenced by their unique and interconnected social positions including, but not limited to, gender, race, class, ethnicity, ability, and age [47]. Moreover, people - including their values, beliefs, and decision making – are shaped by a complex web of interconnected, unequal, and often conflicting interpersonal and societal relationships structured by systemic patterns of power, powerlessness, privilege, or disadvantage operating through colonial, racist, sexist, and ableist power dynamics [47]. A relational lens aims to explore how these structures operate within sociopolitical, cultural and historical contexts to impact the way individuals and social groups exist, develop and engage with one another [47, 48]. Within our study, the use of a relational lens enabled us to reorient our focus away from individual outreach workers’ or participants’ actions dominant in much of the research. Rather, relational theory enabled us to situate our analysis towards their working relationships within the historical and ongoing gendered violence against women experiencing street-involvement, the organization and delivery of care in this community, and both outreach workers’ and women’s perspectives of what constitutes an appropriate and effective working relationship. We further grounded our analysis in equity-oriented principles of harm reduction (Table 1). Specifically, we acknowledged that prohibition, criminalization, stigma, discrimination, housing instability, poverty, and gender-based violence, influence both the types of harms women face and their capacity to navigate and mitigate these harms [49, 50]. Furthermore, in concert with feminist relational theory, we recognize that socio-structural relations of unequal power operate through oppressive social practices to create and sustain the feminization of poverty and the ideologies of deviance concerning women’s substance use practices that ultimately serve to constrain their personal agency [40–42] and negatively impact access and receipt of appropriate care [9, 49, 51]. Our analysis, therefore, sought to illuminate the interconnected dynamics of women’s substance use, their engagement with health care, and their relationships with outreach teams, situated within the larger sociopolitical and historical contexts of their lives.

### Data analysis

STRENTH participant survey data were analyzed using descriptive statistics. Interview data were analyzed using Reflexive Thematic Analysis (RTA) [52, 53]. RTA is an interpretive approach to thematic analysis informed by the theoretical perspectives and researchers’ positionality that underpin the research questions and design [52, 53]. Positionality within RTA requires acknowledging our diverse and shared experiences as a research team as researchers, service providers, and women with lived and living experience of GBV and street-involvement [3]. Thus, as detailed below, our analysis was collaborative attending to the unique strengths and perspectives of team members, and privileging women’s voices. Our interpretation of the findings was embedded in the guidance of our experiential advisory committee to avoid essentialist and stigmatizing language and representation while attending to the structural disadvantage and inequities that women face and their strength and capacities in navigating these concerns.

Themes in RTA are the analytic outputs that represent the patterns anchored by a shared concept within and across the various data sources. In keeping with RTA analytic methods, members of the team (MG, VB, SJ) read and re-read the data noting similarities and differences in how relationships were identified and experienced within and across data sources, particularly in relation to how participants described the nature of their working relationships within the outreach worker – client context. Initially, we created broad codes attending to how people identified the purpose of worker-client relationships, their experiences of positive and difficult interactions, and challenges in building relationships. As coding continued, we attended to the contextual interpersonal and structural circumstances influencing relationships including how outreach services were organized and implemented and how poverty, and historical and ongoing gender-based violence intersected to shape relationships over time. Drawing on our harm reduction perspectives, we further coded for how drug use related stigma and criminalization influenced how relationships were experienced. Finally, we identified patterns within these codes whereby trust emerged as an overarching concept that was embedded in women’s unique experiences of stigma and discrimination in care encounters, the myriad of competing demands they faced in promoting their health and well-being as a direct result of structural inequities, and their overarching desire for improved health and safety in their lives. While multiple data sources were used in analysis, we privileged data gathered through women’s interviews throughout this paper, as our focus was to learn more from women and their experiences engaging with the outreach intervention. We used outreach staff interviews and field notes as a complement to women’s voices, and as a way of providing additional context, especially when this data speaks to specific practices or contextual factors associated with their working relationships with participants. While we recognize the diversity of participants’ social locations (e.g., age, ethnicity, type of disability or health concern) and the historical and ongoing context of colonization and specific impacts for Indigenous women, we drew on the guidance and expertise of the research advisory committee of women with lived experience in how findings and participants are presented. To protect anonymity and respect women’s choices concerning how they were represented in reporting of findings, women were assigned a unique numerical ID versus pseudonyms, and identifying information (i.e., age, ethnicity) is not presented in data excerpts [54]. This approach supported participants’ desire to analyze and present findings that allowed for shared experiences as women in the community.

## Results

### Participants in context

The STRENGTH participants were primarily born in Canada and over half identified as Indigenous (57.9%, *n* = 11); a direct result of the historical and ongoing impact of colonization and associated gendered and racialized violence against Indigenous women in this community [37, 55]. As described in Table 2, concerning participant characteristics, all participants faced unyielding structural disadvantage. Financial strain was severe contributing to food and housing insecurity, and the necessity of what participants deemed “survival strategies” that included, but were not limited to, low-pay volunteer work (e.g., cleaning drop-in spaces), survival sex work, and theft. Slightly more than one quarter of participants (26.3%, *n* = 5) were precariously housed or homeless. Government subsidized housing with varying on-site supports for people living with disabilities was the most common housing. Participants regularly described these spaces as “unsafe”, “filthy,” and/or “without heat, hot water or a private toilet.” While some participants noted housing staff were supportive, fear of eviction and oversights by staff to foster women’s safety at home were shared concerns. All participants discussed living with physical and mental health disabilities and how these conditions, coupled with poverty, limited their mobility and/or capacity to sustain engagement with health and social services. The majority of participants had a primary care provider and a support worker (i.e., case manager), however barriers to care engagement were prevalent contributing to challenges receiving appropriate care. Oversight in service integration was a prominent barrier. Many services for example, were described as failing to consider women’s multiple and interrelated concerns (e.g., chronic diseases such as asthma, diabetes, heart failure; housing or financial assistance applications) thereby forcing women to navigate numerous different organizations or make critical decisions about which service they should prioritize and what needs would remain unmet. Previous negative encounters with service providers where participants felt judged, negated or infantilized exacerbated mistrust and delays in seeking care, resulting in crisis situations requiring emergency services (see Bungay et al., 2025 [4]). In some instances, participants noted being discharged from care or having their access suspended, a situation often associated with being deemed non-compliant by agency staff. The participants’ engagement with health and social services was also deeply impacted by the limited scope of substance use health care in the community. Criminalization of substances, the dearth of timely and appropriate detox, addiction treatment services and safe drug supply, and ongoing violence were notable, contributing to serious harm for instance, withdrawal, drug poisoning, and increased risk of violence as women sought to secure substances and/or were forced to use in unsafe spaces.

**Table 2:** Baseline survey: participant characteristics (*n* = 19)

Participant characteristic	*n* (%)
Age	
Mean (*SD*)	42.6 (12.0)
Range	27–62
Born in Canada	
Yes	18 (94.7)
No	1 (5.3)
Indigenous	
Yes	11 (57.9)
No	8 (42.1)
Housing status^a^	
Housed	14 (73.7)
Precariously housed	5 (26.3)
Financial Strain	
Very difficult making ends meet	8 (44.4)
Somewhat difficult making ends meet	6 (33.3)
Not very difficult making ends meet	2 (11.1)
Not at all difficult making ends meet	2 (11.1)
Current health care provider (*n* = 18)	
Yes	14 (77.8)
No	4 (22.2)
Ease seeing health care provider in last month (*n* = 17)	
Not at all easy	4 (23.5)
Not very easy	1 (5.9)
Somewhat easy	9 (52.9)
Very easy	3 (17.6)
Current support worker (*n* = 17)	
Yes	11 (64.7)
No	6 (35.3)
Ease seeing support worker in last month (*n* = 18)	
Not at all easy	7 (38.9)
Not very easy	1 (5.6)
Somewhat easy	8 (44.4)
Very easy	2 (11.1)
Ability to attend appointments (*n* = 17)	
Not at all easy	3 (17.6)
Not very easy	3 (17.6)
Somewhat easy	8 (47.1)
Very easy	3 (17.6)
Attended ED in the last month (*n* = 18)	
Yes	5 (27.8)
No	13 (72.2)

^a^ “Housed” includes the options: “public, social, or supportive housing,” “supportive SRO,” and “private SRO.” “Precariously housed” includes the options: “couch-surfing,” “shelter,” and “on the streets”

Despite these struggles, participants were proactive in seeking opportunities to enhance their health and wellbeing, noting that their desire to promote and protect their health was the primary reason for participating in the STRENGTH project. Participants reported limited expectations concerning intervention effectiveness citing previous experiences with other outreach programs as rationale. These experiences were described as “feeling like a tick box” for an outreach worker’s paperwork or a loss of authenticity from having to conform or behave a certain way (e.g., be passive and/or act as a victim) in order to receive support. However, the desire to address their needs shaped their willingness to begin engaging and “give the STRENGTH girls a chance” to work with them.

*I was trying to get help. It was right after my best friend died right in front of me and I was really suicidal…and I wanted to get help and needed someone to help me do that…and the nurse in my building is pretty knowledgeable cause they deal with this a lot and they were like ‘OK, we will see if we can hook you up with someone.’ I didn’t have a lot of expectations but then this (STRENGTH) worked out and it was awesome.*

### Building trust: a continuous and iterative relational process

Within these unique contextual features of participants’ lives, trust emerged as the central relational process necessary to foster safe, respectful and strengths-based relationships between women and the outreach teams. Trust was dynamic – building over time – and contingent upon people’s actions, approaches and prior experiences. Participants recounted starting from a place of skepticism and doubt concerning outreach workers’ authenticity and professionalism associated partly with what they described as “betrayal,” “disrespect” and “hurt” with other outreach teams. This uncertainty resulted in testing outreach workers to examine their response to scenarios that had compromised trust in the past:

*Sometimes we learn by harsh manners when we’re on the street. So, you push as hard as you can to have them [outreach worker] break. Well they didn’t break. They didn’t talk about that other person with me when I asked. That was good because it built trust, and I think that’s when it really hit home…if they showed me that they were keeping her stuff private and confidential, then that means they’re keeping me private too.…It’s a test.*

Building and sustaining trust was further imbued with participants’ perspectives about what they deemed the relationships’ “usefulness”. Usefulness was co-created through the collaborative and independent efforts of participants and outreach workers, and whether or not it positively fostered participants’ sense of self-worth. Feeling seen as a person of value who had expert knowledge about their own lives, capable of goal setting and undertaking activities to enhance their health and well-being promoted self-worth. Self-worth grew over time as a result of the participants’ increasing capacities to independently engage with support services. Participants noted previously feeling like “a failure” when navigating services; a sensation associated with fatigue, anxiety, and a general sense of being overwhelmed. Poor health, ongoing violence including assaults by unknown and known men and derogatory and discriminatory encounters with the general public and service providers exacerbated anxiety and fatigue. The effort required to navigate siloed service delivery models added to the sense of overwhelm. However, working at a pace negotiated between the participants and the team, exploring ways to mitigate anxiety and foster their safety, and building upon incremental successes in goal achievement enabled trust in themselves and the team while increasing their confidence and self-worth in moving forward with their goals.

*My main goals were to obtain housing and get healthy. And I have been healthy, because I am not in a wheelchair anymore. It was really good. They would come and pick me up. They were on time and they’d push me to the doctor’s office and stay with me there. and I could get my medications. Now I can go on my own. … And the other thing is like, when I’m depressed, even though I couldn’t come downstairs and there is no buzzer or intercom, when they’re yelling up to me, it would be like ‘hey somebody would care if I died and killed myself.’ … It was really nice that they helped and knowing that somebody thought about me.*

*It was really helpful when I needed it. I had just come out of the psychiatric ward when I met them. I was too anxious to go get groceries, so she took me to the grocery store and helped me buy groceries. It was lifesaving. Now I don’t have as much as a need for services. [Working with them] I just became more confident and less worried about going to doctors’ appointments and more confident and independent.*

### Domains of trust: the “how” of building and sustaining relationships

**Fig. 1:**
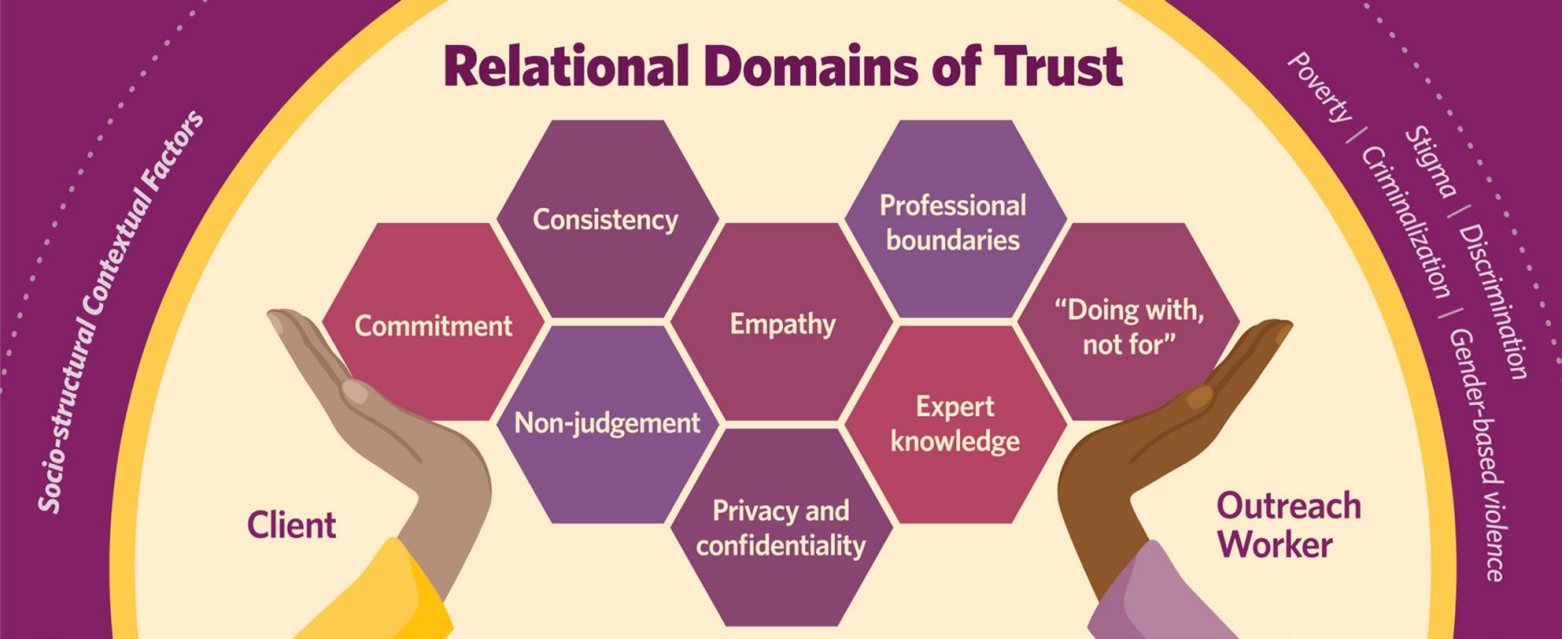
Relational domains of trust

To illustrate the intricate web of relational processes involved in co-creating and sustaining trust, we identified eight interrelated domains (Fig. 1). In the following discussion, we explore these domains, emphasizing the co-creative nature of trust and the critical role of trauma- and violence- informed approaches and outreach workers learning from participants about their experiences with historical and ongoing gender-based violence, poverty, criminalized drug use, limited access to substance use health supports, and other structural inequities. We also demonstrate how engaging with these relational domains of trust can enhance women’s connections with health and social services.

***Commitment***, as a relational domain of trust, included the ongoing and supportive presence of the outreach team; described by participants as “not giving up on me, no matter what.” Commitment was infused with outreach workers’ understandings of the fatigue, anxiety and despair participants felt that, as noted previously, were shaped by the intersecting crises of poverty, violence, poor health, stigma, lack of substance use health services, and criminalization. The ongoing work and emotional distress of navigating these interrelated crises contributed to missed appointments within health and social care and missed connections between participants and the outreach team irrespective of having a set time or location to meet. Participants sometimes “checked out” noting they needed a break from navigating the system. This led to requests for the outreach team to leave or non-response when the teams tried to connect. The outreach team’s consistent check-ins – whether by phone, text, or in person – demonstrated care, which participants responded to by increasing their engagement and setting more complex health-related goals.

*I just needed support. At the time just to talk really, but then it became more about my health. My health started going downhill, in and out of the hospital the past year and she was always there. She visited me in the hospital. … I texted her in situations where I really needed her and she’d be right there, especially in emergency situations. She’d practically drop everything that wasn’t an emergency and be right here for me.*

***Consistency*** in outreach workers and their approach was essential to building and sustaining trust. “High staff turnover” within health and social services was common, and staff and clients noted burnout as a major contributing issue. Additionally, few care spaces provided a dedicated support worker, physician or nurse, leaving participants to “deal with whoever is working that day.” Such inconsistencies contributed to poor rapport, mistrust, and exhaustion associated with having to repeatedly tell their stories which exacerbated incomplete care and a reluctance to re-engage with health and social services. By having a consistent and dedicated outreach team, however, participants noted feeling valued and safe, which was essential to build trust.

*I wasn’t really used to people [support workers] being there in a meaningful way, so it was kind of like, ‘is she just going to be here for [only] a little bit?’ ‘Can I trust her?’ And then she was there every time I needed to talk. The trust just grew more and more.*

Consistency also included the outreach team’s ability to regularly show up at the agreed upon time and place and in a similar mood and level of engagement. Follow-through, meaning an outreach worker would “do what they said they were going to do” and avoid “broken promises” was equally important. These relational dynamics fostered sustained engagement, even when there were times that an unexpected change was required:

*… she never lied to me. If she thinks that she is late, she will say ‘just go ahead without me and I’ll meet you at the place.’ She texts me the address, and says ‘just go, I’m meeting you there.’ It is a good rhythm of how we communicate back and forth.*

***Non-judgment***, as a relational way of engaging, was embedded in outreach workers’ capacities to adhere to the relational elements of harm reduction. Outreach workers conveyed understanding that while managing substance use health was a priority for participants, this was highly individualized and dynamic (e.g., avoiding withdrawal, adhering to treatment interventions) depending on participants’ drug use trajectories. Participants felt respected regardless of their drug use practices, which helped alleviate some of the shame they experienced, especially during periods of increased use. This non-judgmental approach fostered authenticity and honesty in their working relationships, encouraging continued engagement and sustained efforts toward achieving their health and well-being goals.

*It was great to just be human instead of meeting expectations that just aren’t you. With other outreach workers, you kind of have to go, ‘oh no, I wasn’t up all night, I didn’t do that, I don’t do anything illegal; no, I’m totally sober.’ It’s like ugh. With [STRENGTH outreach worker] I can say ‘I’m sorry, I’m a little out of it today. I was up all night. I’ve had a rough week.’ And she is like, ‘it’s okay. We’ll get through this and you can go and catch a nap after.’ You know, she communicates and we just be real.*

***Empathy*** was a co-created experience that required participants and outreach workers to actively communicate, receive, and influence each other’s perspectives during their interactions. It extended beyond the outreach workers’ ability to demonstrate an accurate understanding of participants’ lives; it encompassed a commitment to seeking insights from women about their experiences and using this knowledge to shape their beliefs and actions as outreach workers. Empathy was particularly evident in how outreach workers responded in specific moments, drawing on a shared understanding of a participant’s health challenges and the exhaustion of navigating health or social care systems. Tailoring accompaniments to appointments to address mobility challenges or supporting the rescheduling of appointments when women felt unwell exemplified this empathetic approach.

*If we had something planned and they came by and I wasn’t feeling good, they were very supportive and understanding in knowing that ‘well, we don’t have to do it today.’ Because with disabilities and chronic health problems, you have to be really understanding of that. Some days you just don’t know. [They were] completely understanding … Just being able to do it another day was helpful.*

Facilitating and maintaining participants’ ***privacy and confidentiality*** as research participants was also important. Participants shared numerous accounts of threats to their privacy and confidentiality in daily life, including police surveillance, the high public visibility associated with street-involvement, and the lack of discretion in crowded care spaces. These experiences were often described as “violating” and “dehumanizing.” For participants, privacy and confidentiality with the STRENGTH team meant that personal details, goals, activities, and progress in the project were not disclosed to others. Additionally, conversations took place in private spaces whenever possible and only with the participant’s consent. While participants were not concerned about their engagement with STRENGTH being publicly known, they expressed unease about the gossip that frequently circulated among community staff and other residents.

*And our anonymity, they keep that very private. … Because my friend is part of the project too. They don’t discuss her with me at all. And I know it would go the same way so that’s good. … That was part of building the trust with me, is that I know that they’re committed to our anonymity and confidentiality.*

Privacy and confidentiality were further co-constructed through the ways women and outreach workers navigated interactions with other support services. Participants frequently encountered health care providers in public spaces, where discussions about treatment plans or follow-ups provided little privacy regarding their health concerns. Trust was strengthened when outreach workers acknowledged these challenges and facilitated care in private settings. Outreach workers also actively promoted privacy and confidentiality during care encounters by holding care providers accountable when unnecessary questions about participants’ private lives were raised.

***Professional boundaries*** were essential and required ongoing communication and negotiations between participants and the outreach team. They collaboratively navigated the tension between the extreme loneliness and isolation participants experienced and established realistic boundaries and expectations of the nature and scope of their working relationships. Detachment, described as focused solely on a task and not feeling valued as a person, was antithetical to trust. The participants preferred a working relationship where they experienced a sense of connection, felt cared for, and that they had someone “they could rely on.” Participants also recognized and valued the workers’ maintaining boundaries about sharing in their personal lives.

*When we meet up, she was happy to see me and it was a break from whatever I had going on. She never told me anything too much about her own stuff, because of the whole professional thing, which I appreciated, but it was like we were friends in some way. It’s not that she was more than my worker, but I looked forward to seeing her and you could tell that she looked forward to seeing me too. We got things done, and it was fun doing it.*

Co-creating boundaries around outreach workers’ availability and time constraints was essential. Participants felt a sense of connection even during periods of intermittent contact, such as when a worker was on holiday. Although some participants occasionally struggled with limited worker availability due to workload, their trust was sustained through transparent and reliable communication.

*I saw her on the street and she couldn’t talk right then, I felt a little bit hurt. I was okay but then she said she’ll talk to me after. That was okay then. … I just told her I felt hurt. I texted her and told her that, and then she talked to me. We would work through it. They understood me by the way they talked to me. … Nice, friendly, always caring. You can tell they care about people.*

***Expert knowledge*** functioned as a relational process involving the co-creation of shared understanding between participants and outreach workers. This encompassed both parties’ experiential knowledge and how it was applied to support participants in achieving their health and well-being goals. Participants were recognized as experts of their own care needs, including their strengths in managing these needs and the barriers they faced in accessing timely, appropriate care. Outreach workers contributed specialized knowledge of local services, eligibility criteria, safe care environments, and system navigation skills. Rooted in a mutual commitment to collaboration, participants’ trust in the outreach team grew as they experienced the workers’ efficiency and skill in navigating complex systems to facilitate care engagement. Tailoring this expertise to each individual required time and ongoing dialogue to foster a shared understanding and effective application of knowledge.

*She was really knowledgeable and got me to the right services and the right kind of people. She kind of knew. It was a pretty long period of time [we worked together], so obviously she kind of had me figured out [because she took the time]. She knew where to take me and who I’d get along with and all that.*

Sharing this expert knowledge fostered sustained trust, enabling long-term planning and anticipation of future needs. Outreach workers were recognized as knowledgeable intermediaries who bridged access to otherwise inaccessible services and were trusted to accurately translate and communicate participants’ needs and concerns when necessary.

*She’s my mouthpiece. It’s important for me to have a mouthpiece I trust. You know, when you come to Canada, some people don’t understand how I speak. And the motive of people here, if she understands, she will tell you no. Definitely she will tell you no. … It has given me peace and comfort because … at least I have somebody who will explain for me better and defend me, and she also can tell you what type of a person I am.*

***“Doing with***,*** not for”*** was a complex and deeply relational process that strengthened participants’ trust by demonstrating the team’s respect for women’s rights to self-determination and recognition and support of their unique strengths and capacities to manage their health and well-being. This approach involved attending to and building upon participants’ strengths with the goal of fostering greater independence in managing their health. Participants viewed this capacity-building approach as a direct contrast to clinical encounters where they often felt infantilized by paternalistic practices that overlooked their perspectives and care needs. By embracing “doing with,” outreach workers and participants built trust that enabled open, sometimes difficult conversations – such as discussing the consequences of postponing appointments or expressing frustration toward care providers.

*They reminded me all the time what my goals were because I would tend to forget or try to push them off sometimes, like with my depression I’d try to forget about it and not do it or whatever. But she was like ‘this is a positive thing, this is what you wanted, remember what you wanted?’ … that’s what helped me, pushed me to get there and like, ‘okay give me ten minutes and I’ll be out of bed, let’s go.’*

Participants described the exceptional challenges of exercising agency within systems marked by stigmatization, discrimination, and harm. The outreach workers’ approach of “doing with, not for” fostered trust by supporting women’s active collaboration to assert their agency within these spaces, reinforcing their right to respect and confidence in their ability to act in their own best interests.

*I guess it’s my own struggle, but I do need support because I’ve been in the psych ward a lot and I’ve been around that patronizing attitude, so it helps me to talk [with the workers], it helps me to show somebody that I’m bright, I make my own decisions, I’m not just a taker; I like to show my humanity and they help me to be human even though I struggle with certain mental health things.*

This sense of agency was highly motivating and helped participants maintain their commitment to “better days and holding it together” despite obstacles encountered during care engagement. Ultimately, women reported an increased sense of motivation grounded in this trust and collaboration.

## Discussion

This study sheds light on the complex and relational dynamics of outreach interventions among women experiencing street-involvement, focusing on trust as a central process underpinning engagement with health and social services. Our findings underscore the profound impact of intersecting structural inequities – including colonization, poverty, gender-based violence, and drug criminalization – on women’s health experiences and access to care. These socio-structural factors shaped participants’ prior service encounters, frequently characterized by distrust, stigma, and marginalization, which influenced their initial skepticism toward outreach efforts. Similar to other studies [19, 22, 25, 29, 56] we illustrate the importance of trauma- and violence-informed, strengths-based, and respectful approaches that foster psychological safety to promote engagement with outreach teams. Our findings, however, expand upon this work to illustrate the complexity of trust, noting how eight interrelated domains are enacted and experienced, and how trust serves as a critical foundation for enhancing women’s engagement with services, ultimately supporting their autonomy, well-being, and personal capacities to manage their health amid these complex structural barriers.

A key contribution of this work is the identification of trust as a dynamic, co-constructed and iterative relational process that evolves over time through collaborative and strengths-based interactions with consistent outreach teams. As has been reported elsewhere [51, 57, 58], trust was not automatic or static. Trust required outreach workers to enact commitment and respect for privacy, while maintaining professional boundaries that aligned with participants’ expectations and capacities for engagement on any given day. Additionally, while empathy is typically considered unidirectional, with service providers demonstrating understanding of a client’s circumstances, our findings echo those of van Dijke et al. [59], demonstrating empathy as co-creative and dynamic process. Trust relational domains were instrumental in fostering a safe environment where women could engage authentically without fear of judgment or breach of confidentiality. The findings align with broader literature emphasizing a relational approach to trust and the critical role trust plays in health care engagement for people affected by systemic inequities generally [60, 61], and among women who use unregulated drugs and are impacted by gender-based violence, discrimination and criminalization specifically [12, 51, 57, 62].

Our findings also reinforce feminist [7–9, 47] and other critical scholars’ [11, 25, 63–65] critiques of individualistic and blaming narratives concerning care engagement that overlook socio-structural barriers to care. Informed by relational perspectives of engagement, harm reduction and violence, our findings conceive that trust building takes place amid significant systemic inequities operating in women’s lives [47]. For instance, in examining the elements of trust, a non-judgmental approach involved countering hegemonic discourses of abstinence and deviance that are stigmatizing and discriminatory, while simultaneously aiming to interrupt systemic patterns of power in care encounters. Such an approach required integration of expert knowledges of both outreach workers and participants, which in turn also enabled outreach workers to function as a critical bridge to services that are otherwise fragmented and difficult to access. Working from women’s self-identified priorities and engagement readiness, this role as trusted intermediary facilitated practical support and enhanced participants’ confidence and self-worth, enabling greater capacity in managing their health and social care needs. It is well established that knowledge co-sharing and collaboration are essential to meaningfully address women’s inequities in health and in appropriate health care [12, 66], and our findings add to this evidence-base by illustrating the intersections between trust and client-led approaches to care.

Our data also provides further support that trust-building in care engagement often begins from a place of skepticism rooted in past experiences of “betrayal” and disrespect by service providers [5, 9, 62, 67]. Participants’ “testing” of outreach workers reflects the high stakes involved when past breaches of trust have compromised care. The success of the STRENGTH intervention, therefore, hinged on outreach workers’ capacity to withstand this scrutiny, demonstrating reliability and genuine care. This collaborative approach, which centers women’s strengths, expertise and rights to self-determination and recognizes their motivation and desire for enhanced health, contrasts starkly with traditional paternalistic models of care and/or outreach. Thus, these findings add to the growing body of evidence of outreach effectiveness with strengths-based versus deficit-oriented approaches to engagement [21, 22]. Importantly, this study foregrounds the impact of intersecting oppressions such as poverty, gender-based violence, health inequities, and other forms of structural disadvantage (e.g., racism) as critical to inform dimensions of outreach engagement. As trauma- and violence-informed approaches provide critical insights into the complex interplay of individual and collective trauma including the experience of these oppressions historically and in participants’ current contexts, while also promoting safety and empowerment [3, 44], outreach efforts that recognize and respond to these intersecting experiences are more likely to foster trust and meaningful engagement [4, 69].

The findings from this study have several critical implications for drug policy and service provision targeting women experiencing street-involvement. First, policies should promote outreach programs that emphasize trust-building through consistent, respectful, and empathetic engagement. Funding and program design must support outreach workers in developing long-term relationships that acknowledge women’s lived expertise and autonomy rather than relying on punitive or transactional approaches [58, 69]. Given the disproportionate impact of colonization and systemic inequities for women marginalized by poverty, sexism and racism, policies must mandate culturally safe, trauma-informed, and gender-specific services. This includes further work to consider how to embed anti-racist and anti-colonial frameworks within service delivery, while simultaneously exploring the diversity of the workforce necessary to foster trust among women of diverse social identities [68]. Additionally, criminalization, housing instability, and fragmented health care are consistent structural barriers impeding engagement. Policies must focus on expanding and streamlining accessible substance use treatment, harm reduction, housing supports, and mental health services tailored to women’s needs [2, 8]. Effective policy must extend beyond health services to include housing, justice, social services, and community-led initiatives. Coordinated cross-sector strategies are needed to address poverty, violence, and criminalization that underpin women’s marginalization and impact service engagement. Investing in specialized training for outreach workers on trauma- and violence- informed care, cultural humility, harm reduction, and gender-based violence is essential to maintain quality relational care [67]. Policies should recognize and compensate outreach workers adequately, reflecting the complexity and emotional labor involved in their roles [23]. Meaningful involvement of women with lived experience in the design, implementation, and evaluation of outreach programs and policies will ensure responsiveness to their needs and priorities, enhancing the relevance and effectiveness of interventions [3]. In sum, policy reforms must move toward holistic, equity-driven frameworks that prioritize relational trust, gender equity, cultural safety, and structural transformation. Such shifts are critical to improving health outcomes and social inclusion for women facing intersecting vulnerabilities associated with street-involvement and substance use.

While this study offers in-depth insights into the relational processes involved in building trust within outreach settings, certain limitations warrant consideration. The findings are specific to women engaged with the STRENGTH outreach team and may reflect the unique context and dynamics of this particular project. Additionally, participants who volunteered for the study may have had particular experiences that shaped their willingness to participate, which could influence the range of perspectives captured. Moreover, the complex, evolving nature of trust means that some nuances might not have been fully captured within the scope of this research. Finally, while our findings did not highlight differences experienced by women based on specific gender or other social identities, further work to nuance the unique and shared culturally specific aspects of outreach is warranted. Future studies could explore these relational processes over time, across varied contexts and social identities to enrich understanding.

## Conclusion

Taken together, the activities of co-building and sustaining trust enabled participants and outreach workers to co-create knowledge and understanding; collaboratively build capacity and co-facilitate and navigate health and social care. Despite the promising relational processes observed however, persistent structural barriers such as limited substance use treatment options, criminalization, and fragmented care systems continue to impede women’s access and sustained engagement. These findings reinforce the urgent need for health and social policies that address systemic inequities and expand comprehensive, accessible, and culturally appropriate services for women living at the nexus of poverty, violence, health inequities, and unregulated substance use.

## Supplementary Information

Below is the link to the electronic supplementary material.

Supplementary Material 1: Qualitative interview guides

## Data Availability

The datasets generated and/or analysed during the current study are not publicly available due to the highly sensitive nature of participants’ data and on recommendation from the community advisory committee. Participants in this study did not provide consent to have anonymized data available for secondary analysis and therefore data is not available. The qualitative interview guides are published as supplementary material.
