# Supplementary material for: Relational trust in outreach with women experiencing street-involvement in British Columbia, Canada: a qualitative study
Source: BMC Health Serv Res. 2025 Dec 11;26:71. doi: 10.1186/s12913-025-13875-3 (PMC12801862; doi:10.1186/s12913-025-13875-3)
Supplement: Supplementary file 1 — Supplementary Material 1: Qualitative interview guides [file 12913_2025_13875_MOESM1_ESM.docx]

**Supplementary Material**

**Qualitative Interview Guide (Women)**

1. To get started, it would be helpful if you could tell me a little bit about how you first learned about this project. For example, can you tell me how you first learned about the STRENGTH program? How did you meet people from this team? Some people find it helpful to think about the first time they learned that this program existed or first met one of the outreach team. Can you tell me more about that please?
2. In a program like this, we know that every woman is unique and might engage with the outreach team in their own way and for different reasons. Please tell me about how you decided to work with the team.
3. These next few questions focus on your ongoing relationship with the team and goals that you had that you hoped that the team could help you with.
   1. Please describe for me your main goals that you had working with the project? It might be helpful to just think of one or two if that is easier.
   2. Did the goals that you set feel like you could achieve them?
   3. Did you feel you could meet your goals in a reasonable amount of time?
4. Please describe for me what you think were the most positive things about working with this study for you.
5. Please describe for me if you experienced any type of challenges working with the project team to obtain the goals you were hoping to achieve.
6. Overall, has working with project been helpful for you?
7. From the beginning of your engagement with the project, what percent of your goals/expectations were met? Are you satisfied with what you achieved in the project?
8. What was your experience working with the project outreach teams in comparison to working with other outreach teams?
9. What do you think we need to communicate about this program to other service organizations in the DTES? What do you think they could learn from your experience?
10. What do Outreach Workers really need to know about this community before they come to do this work? What do they need to understand about how to work with women in this neighbourhood?
11. What other thoughts or ideas do you have about this project? For example, is there anything else you think we should know about to better support women to engage with services in the DTES?

**Qualitative Interview Guide (Service Providers and Outreach Workers)**

1. Please tell me a bit about yourself and your role at your organization.
2. Please tell me about who your organization serves
3. Please describe a ‘typical day’ day for you.
4. This study aims to better understand the outreach needs of highly marginalized women living in the DTES who have been affected by violence. By marginalized we mean women who in addition to violence may be experiencing mental health and addictions issues and structural vulnerability like homelessness. What is your experience working with this group of women?
5. Please describe what role you think outreach has in supporting highly marginalized women in the DTES.
6. Please describe what role you think outreach has in supporting the work of health care and service providers in traditional settings (e.g., clinic or agency). For example, describe the working relationships with health care and service providers in your organization and outside your organization. What are the referral procedures?
7. Please describe any recommendations you might have for how providers in traditional roles can better utilize outreach workers.
8. Do you have any suggestions about how improve outreach services for highly marginalized women in the DTES?
9. Do you have any suggestions about how you or other organizations can better support outreach services for highly marginalized women in the DTES? For example, how would you improve the integration and collaboration of outreach workers and their work with other services and systems, and funding.
10. Is there anything else you would like to share with us about services for women in the DTES.
